# Visual and auditory synchronization deficits among dyslexic readers as compared to non-impaired readers: a cross-correlation algorithm analysis

**DOI:** 10.3389/fnhum.2014.00364

**Published:** 2014-06-10

**Authors:** Itamar Sela

**Affiliations:** Edmond J. Safra Brain Research Center for the Study of Learning Disabilities, University of HaifaHaifa, Israel

**Keywords:** dyslexia, asynchrony, crossmodal integration, EEG, ERP, cross-correlation analysis

## Abstract

Visual and auditory temporal processing and crossmodal integration are crucial factors in the word decoding process. The speed of processing (SOP) gap (Asynchrony) between these two modalities, which has been suggested as related to the dyslexia phenomenon, is the focus of the current study. Nineteen dyslexic and 17 non-impaired University adult readers were given stimuli in a reaction time (RT) procedure where participants were asked to identify whether the stimulus type was only visual, only auditory or crossmodally integrated. Accuracy, RT, and Event Related Potential (ERP) measures were obtained for each of the three conditions. An algorithm to measure the contribution of the temporal SOP of each modality to the crossmodal integration in each group of participants was developed. Results obtained using this model for the analysis of the current study data, indicated that in the crossmodal integration condition the presence of the auditory modality at the pre-response time frame (between 170 and 240 ms after stimulus presentation), increased processing speed in the visual modality among the non-impaired readers, but not in the dyslexic group. The differences between the temporal SOP of the modalities among the dyslexics and the non-impaired readers give additional support to the theory that an asynchrony between the visual and auditory modalities is a cause of dyslexia.

## Introduction

According to a widely accepted definition of developmental dyslexia, a dyslexic reader is one who exhibits slow and inaccurate reading performance unrelated to his/her IQ level or educational opportunities (British Psychological Society, 1999; Lyon and Shaywitz, 2003; Shaywitz and Shaywitz, 2008). An abundance of research into phonological deficits has indicated that the primary source of the difficulties experienced by dyslexic readers lies in word decoding accuracy (Liberman and Shankweiler, 1991; Badian, 1997). Some studies have found that disabled readers demonstrate a fundamental orthographic deficit (Stanovich and West, 1989; Cunningham and Stanovich, 1990; Zecker, 1991; Barker et al., 1992; Morris et al., 1998). The reading deficits of developmental dyslexia persist into adulthood (Bruck, 1992; Leonard et al., 2001). A large number of studies have shown deficient phonological processing as a core deficit in developmental dyslexia. The leading theory, the phonological deficit theory of developmental dyslexia (Stanovich, 1988; Share, 1994; Snowling, 1995), suggests that dyslexic readers may suffer from an (unspecified) dysfunction in peri-sylvian brain regions, which leads to difficulties in generating and processing accurate and efficient phonological representations of speech sounds (Stanovich, 1988; Temple et al., 2001).

In addition, researchers are still debating whether dyslexic readers are characterized by impairment in basic auditory and/or visual processing (Amitay et al., 2002a,b; Vellutino et al., 2004; Lachmann et al., 2005; Groth et al., 2011). In the auditory domain, data has indicated dyslexics' inability to discriminate between temporal rapidly changing tones and consecutive acoustic events (Tallal, 1980; Tallal et al., 1993, 1998). Difficulties locating the origin of sounds and blending them were also found (Stein, 1993). In the visual domain, findings have indicated that dyslexic readers have smaller and fewer neurons in the magnocellular layers of the Lateral Geniculate Nucleus (LGN) (Livingstone, 1991) suggesting fundamental deficiencies of the magnocellular system and pathways of the visual cortex that are responsible for motion, contrast sensitivity (Stein and Walsh, 1997; Stein and Talcott, 1999; Stein, 2001; Stein et al., 2001) and fast sequential processing (Ben-Yehudah and Ahissar, 2004). Based on the aforementioned studies regarding the presumed low-level visual and/or auditory sensory deficit among dyslexic readers, it was recently demonstrated, using the Pair Associate Learning paradigm (Hulme et al., 2007), that dyslexic readers exhibited a crossmodal association difficulty (Jones et al., 2013). Previous data (Breznitz, 2002, 2003, 2006) has found that the gap in the speed of processing (SOP) between the visual and auditory modalities is wider among dyslexic readers than non-impaired readers. This gap prevents the precise integration over time (Berninger, 1990; Wolf and Bowers, 2000) of the crossmodal information necessary for accurate word decoding and leads to the asynchrony phenomenon. Breznitz (2006) suggested that the asynchrony phenomenon in the word-decoding process occurs when there is time gap resulting in a mismatch between the grapheme and its phoneme correspondent.

It was previously suggested that different frequency ranges are important to speech perception (Luo and Poeppel, 2007; Ghitza and Greenberg, 2009). The temporal sampling framework for developmental dyslexia (Goswami, 2011) proposes that at the basic level of auditory perception processing, dyslexic readers have difficulties distinguishing between different frequency ranges, an impairment that leads to a slower and less accurate speech perception (Goswami et al., 2010). It is reasonable to assume that an impairment in sensory temporal processing that can be identified at the frequency domain may be also expressed at the time domain. Thus, it is important to study whether dyslexic readers exhibit an abnormal basic sensory (auditory or visual) information processing. Here, the dyslexic readers' ability to process fundamental sensory input is investigated by using a time-based cross-correlation analysis.

The synchronization hypothesis proposes that for accurate information processing to occur, it is crucial that the information that arrives from more than one modality be integrated in both content (Fujisaki and Nahida, 2005; Ghajar and Ivry, 2008; Neil et al., 2011) and time (Llinas, 1993; Breznitz, 2001, 2002, 2003, 2006; Breznitz and Misra, 2003; Breznitz et al., 2013). The act of reading relies on the information processing system and during the word decoding process both the visual and the auditory modalities are activated. Word decoding accuracy can be achieved only when the activation within and between modalities is synchronized (Breznitz, 2008). It was found that the time gap between the visual and auditory Event Related Potential (ERP) component of P1 of the dyslexic readers was larger than 100 ms, whereas the non-impaired readers exhibited an insignificant time gap of 15–30 ms only (Breznitz, 2008). A similar trend of results was found when the time gap between the auditory and visual ERP of N1 was analyzed. Moreover, it was shown that the between-modalities time gap has developmental constraints: while the auditory components of N1 and P1 had shorter latency as compared to visual ones among dyslexic children, the opposite was found among adult dyslexic readers (Breznitz, 2008). The author argued that these results support the notion that a deficit within the visual modality affects decoding ability, a claim that was supported by evidence of a significant correlation between the between-modalities time gap and reading performance (fluency and reading errors).

The visual-orthographic and auditory-phonological systems are the core systems activated temporally during the word-decoding process. However, during a normal course of processing, both systems differ not only in the structure and length of their neural networks, but are also located in different parts of the brain (Saito et al., 2005) and operate in a different manner and at different speeds (Breznitz, 2002, 2003, 2006). Data has indicated that auditory information arrives (Mishra et al., 2007) at the auditory cortex, about 30 ms after stimulus presentation (Heil et al., 1999), whereas visual information arrives (Mishra et al., 2007) at the visual cortex about 70 ms after stimulus presentation (Schmolesky et al., 1998). This suggests that when a stimulus includes both visual and auditory representations, the auditory stimuli arrive in the brain faster than the visual stimuli. However, at the word-decoding level, the auditory-phonological channel perceives and processes the information in a temporal-serial manner (Rosenzweig and Bennet, 1996), whereas the visual-orthographic channel process information in a spatial, holistic manner (Breznitz, 2006). This suggests that visual processing at this level might be faster than auditory processing. Yet, an effective word decoding process requires an exact integration of graphemes and phonemes (Adams, 1990; Berninger, 2001). In other words, the two systems need to be synchronized for appropriate crossmodal integration to occur. The focus of the current study is to quantify the synchronization of the two modalities among non-impaired and dyslexic readers.

Earlier studies that verified the relationships between visual and auditory processing among non-impaired readers suggested that presenting a visual pattern prior to an auditory one eases inter-sensory correspondence while the presentation of an auditory pattern prior to a visual one increases correspondence errors (Botuck and Turkewitz, 1990). It has been suggested that either the information might be received more accurately through the visual as opposed to the auditory modality, or it is more difficult to register information arranged temporally than information arranged spatially in memory (Botuck and Turkewitz, 1990). Furthermore, Ben-Artzi and Marks (1995) examined whether and how stimulus type influences visual-auditory interaction. In Ben-Artzi and Mark's study (1995), participants were asked to classify sound levels and spatial locations on two types of tasks: uni-dimensional and bi-dimensional. Data indicated that visual identification is not only faster (see also Melara and O'Brien, 1987) but also dominant (Egeth and Sager, 1977) over the auditory identification. Posner et al. (1976) suggested that visual dominance is, in fact, a compensation for the visual system's limited capacity to arouse internal attention. According to this approach, the increases in sound level in the auditory system arouse attention automatically. However, the arousal of attention in the visual system requires specific controlled effort and the brain learns to allocate attention to visual stimuli. Furthermore, it has been suggested that the existence of the visual system, which can allocate attention to spatial stimuli, enables the ear to relate to its relevant stimuli (Posner et al., 1976). By using fMRI imaging techniques, recent studies have indicated that during crossmodal activation, when both visual and auditory information are presented, the visual modality is dominant at the pre- response level whereas the auditory is more dominant at the response level (i.e., Koppen et al., 2009; Chen and Zhou, 2013). Thus, all the above support the notion that a deficit in the visual modality might be involved in dyslexia. In light of the assumption that the visual modality has dominancy over the auditory modality, it is important to investigate the relationship and interaction between the two sensory modalities.

The basic assumption of this study is that exposing a participant to either a visual or an auditory stimulus triggers sequential information processing which has an effect on ongoing ERP activity. Furthermore, visual-only or auditory-only information processing is carried out in a similar fashion irrespective of whether the stimulus is solely visual or accompanied by an auditory stimulus presented at the same time (crossmodal presentation). Therefore, the first hypothesis of this study is that the ongoing ERP of the crossmodal condition will contain visual and auditory elements that will also be found in each of the unimodal conditions (Marchant and Driver, 2013). It should be noted that the current study does not assume that an apparent correlation between the unimodal and crossmodal ERPs stems solely from the presence of the unimodal element within the crossmodal ERP. It is proposed that to a certain extent, the two datasets share a common factor which may be explained as a specific unimodal brain reaction.

The second hypothesis asserts that although the information processing of a unimodality (specific-visual or auditory) will be similar under the two conditions of the unimodal stimulus type and under the two modalities stimulus type, the crossmodal processing will affect the SOP of the uni (specific) modality. Thus, if the data were to be looked at in an individual time window for the unimodal condition, it is assumed that a similar pattern of the component (factor) will be found in a corresponding time window for the crossmodal condition, but not necessarily in exactly the same time location, as it may appear earlier or later [this is defined as Delta Time (DT)]. The polarity of DT indicates either an acceleration (negative DT) or deceleration (positive DT) in one modality's SOP as a result of the presence of the second modality.

As the SOP of the two different modalities was shown to differ (Saito et al., 2005) the third hypothesis of the current study is that differences will be found between the size of the interaction between the visual and auditory modalities and the influence of one modality on the other. Moreover, as dyslexic readers' ability to process uni (visual or auditory) and crossmodal sensory information was found deficient (Lachmann et al., 2005; Breznitz, 2006; Jones et al., 2013), the fourth hypothesis of the current study is that differences between the two-reading-level groups will be found. Specifically, it is assumed that the results of the current study would show a negative effect of one modality (visual or auditory) on the SOP of the other modality among the dyslexic readers.

## Methods

### Participants

Nineteen dyslexic readers (age 25.5 ± 2.91) and 17 non-impaired readers (age: 24.52 ± 2.29) were included in the study [*t*_(35)_ = 0.191, *p* = 0.242]. None of the participants had a history of neurological or emotional disorders, and no differences were found between dyslexics and non- impaired readers in attention ability as measured by the d2 test for adults [*t*_(35)_ = −0.222, *p* = 0.825] (Brickenkamp, 1981). The dyslexic readers were diagnosed with dyslexia during childhood, and their diagnosis was confirmed as adults by achieving one standard (−1) score or below on the Hebrew “MATAL” normative reading test (MATAL Battery, 2007). The non-impaired readers were recruited via notices posted on campus bulletin boards. Individuals with dyslexia were recruited through the Student Support Service at the University of Haifa. All participants were native Hebrew speakers from a middle-class background, right-handed, and screened for normal hearing. All participants displayed normal or corrected-to-normal vision in both eyes. All participants gave their informed written consent prior to inclusion in the study, and all were paid as compensation for their time. The experiment was approved by the University of Haifa Ethics Committee (Number, 1991) according to the Helsinki Declaration.

The classification of participants into non-impaired readers and dyslexic readers groups was based on the following behavioral measures (For more details see Breznitz and Misra, 2003; Breznitz et al., 2013).

#### Intelligence

Intelligence was tested by the *Block Design* (performance) and the Similarities sub-tests (verbal) from the WAIS-III (Wechsler, 1997).

#### Decoding skills

One Minute Tests (Breznitz and Misra, 2003) comprised a battery of two subtests one for words and the other for pseudowords in which the participants were asked to read single words or pseudowords as quickly and as accurately as possible within the space of 1 min.

#### Reading rate and accuracy of connected text

Oral reading time and accuracy of a narrative text comprising 247 words (MATAL Battery, 2007. See also Breznitz and Misra, 2003). Reading time was defined as the mean reading time for each word read correctly.

#### Reading rate and comprehension

A Reading Comprehension Test (MATAL Battery, 2007), comprising 412 words. The participants were asked to silently read a passage as quickly as possible and then answer 18 comprehension questions. Reading time was based on the mean reading time per word. Comprehension scores were based on the total number of correct answers.

#### Memory

In the Digit Span (WAIS III, Wechsler, 1997), the standard scores of each participant were recorded.

#### Speed of processing (SOP)

Two tests were used to assess SOP: The Digit Symbol Task and Coding Task-Speed Factor (WAIS III, Wechsler, 1997).

Table 1 presents the means, standard deviations, and *t* values for the reading and the cognitive background measures. The dyslexic readers achieved significantly lower scores than the non-impaired readers in reading accuracy and time parameters but not for silent reading comprehension. In addition the dyslexic readers also obtained significantly lower scores compared to the non-impaired readers in the SOP and working memory measures but not in the intelligence measures (see Table 1).

**Table 1:** **Reading and cognitive measures for dyslexic and non-impaired readers**.

	**Non-impaired readers group**		**Dyslexic readers group**		***t***
	**Mean**	***SD***	**Mean**	***SD***	
One minute test—number of words read correctly	111.04	16.99	65.63	20.71	−8.87^***^
Pseudowords per minute—number of correct Pseudowords read	59.89	15.68	27.50	11.05	−8.70^***^
Oral reading (per letter reading rate)	0.69	0.18	0.41	0.09	6.19^***^
Silent text reading rate—total reading time	212.05	54.69	128.83	30.12	−6.89^***^
Reading comprehension (correct answers out of 18 questions)	16.67	2.40	15.54	4.70	1.06
Digit symbol SD score	12.60	10.75	8.23	15.16	−2.63^**^
Coding SD Score	13.87	10.86	8.90	5.98	5.57^***^
General ability similarities	12.01	3.86	11.90	2.91	1.57
Block design	12.67	2.29	12.85	1.66	1.21
Digit span—standard score	12.42	3.08	9.00	2.76	−4.38^***^

** p < 0.01;

*** p < 0.001.

### Apparatus

Two computer sets were used in this study. The first computer was used to present the task stimuli (visual as well as auditory, using a screen, and a pair of speakers) and to record participant's responses. The electrophysiological data was collected using a Bio-Logic Brain Atlas IV computer system (2nd computer set) with 20 electroencephalographic (EEG) activity reception channels. The data collection from the scalp began at approximately 1000 ms prior to the beginning of the experimental task. The sample rate was 256 Hz and was carried out using a full array of electrodes placed according to the International 10/20 system (Jasper, 1958) utilizing an Electro-cap (a nylon cap fitted over the head with 9 mm tin electrodes sewn within). An electro-oculogram (EOG) was recorded with an electrode extension that was located under the left eye. A ground electrode was placed on the left mastoid. All electrodes were maintained at an impedance of 5 KΩ or less. Brain activity was sampled directly from 19 scalp electrodes (Fp1, Fp2, F7, F3, Fz, F4, F8, T3, C3, Cz, C4, T4, T5, P3, Pz, P4, T6, O1, O2).

### Task design

A visual, auditory, and crossmodal processing task (Breznitz and Misra, 2003; Meyler and Breznitz, 2003) was administered. This task consisted of 150 stimuli presented to the participants in three different conditions: auditory alone (50 tones occurring at 1000 Hz with a time length of 200 ms), visual alone (50 white rectangle shape stimuli, presented at the middle of a black screen for duration of 200 ms), and crossmodal (50 tones and flashes occurring simultaneously). The 150 stimuli were presented in a randomized order. The between trials interval, i.e., the time from the beginning of one trial to the beginning of the next trial was set to 2 s. The participants were asked to press one of three computer keys—One key to indicate the appearance of an auditory tone stimulus alone, one key for the visual rectangle-like flash, and the another key to indicate when the two stimuli occurred simultaneously. Off-line analysis differentiated between the auditory, visual, and simultaneous segments. All stimuli were presented to the participants on a PC computer. Participants were seated 0.5 m from the computer screen and heard the tones via speakers.

### Procedure

During data collection, participants were seated in a sound attenuated room. The experiment took place during two sessions of about 2 h each. The first part of data collection consisted of gathering the behavioral measures, and in the second part the experimental tasks ERP measures were incorporated.

### Data analysis

For each participant and for each of the three task conditions, both the mean value of Reaction Time (RT) for correct responses and accuracy were computed. The EEG data were segmented into 1945 ms epochs (one per trial) and by three different conditions based on the trial type (1-visual-only, 2-auditory-only, 3-crossmodal stimulus). Data of one trial started 445 ms prior to stimulus presentation and ended 650 ms following presentation. The data was corrected for eye movements using the Orgil Medical Equipment (1997), normalized by comparing cognitive activity time with inactive time for each electrode, separately, for each participant, filtered using a low pass 20–24 Hz filter, and averaged by stimulus type (Orgil software, 1997) prior to beginning the cross-correlation analysis.

### Cross-correlation analysis

In order to verify the research hypotheses, a cross-correlation analysis was obtained. Cross-correlation analysis is a procedure used in signal processing by which the similarity between two signals is measured. The algorithm analysis output is a series of correlation coefficients between two signals according to time, i.e., one correlation coefficient is calculated for successive time points according to a specific temporal increment (Woody, 1967; Nelson-Wong et al., 2009). Previously, the cross-correlation technique was used as part of a tool aimed at overcoming the limitations of the traditional ERP averaging method and to categorize participants based on their ERP data (Sela et al., 2008). Similar to the work of Sela et al. (2008), the current study algorithm obtained and compared data time windows taken from participants' ERPs. These data time windows included all 19 electrodes within the selected time areas. In the following algorithm, the term “unimodal” refers to either visual- or auditory-only ERP datasets. The term “crossmodal” refers to the synchronized visual-auditory stimulus's ERP.

### Time window analysis—creation of a cross-correlation graph

The following algorithm was used on three different datasets: visual-only ERP, auditory-only ERP, and crossmodal ERP. The explanation below describes the process in which a cross-correlation graph is computed.

**Figure 1:**
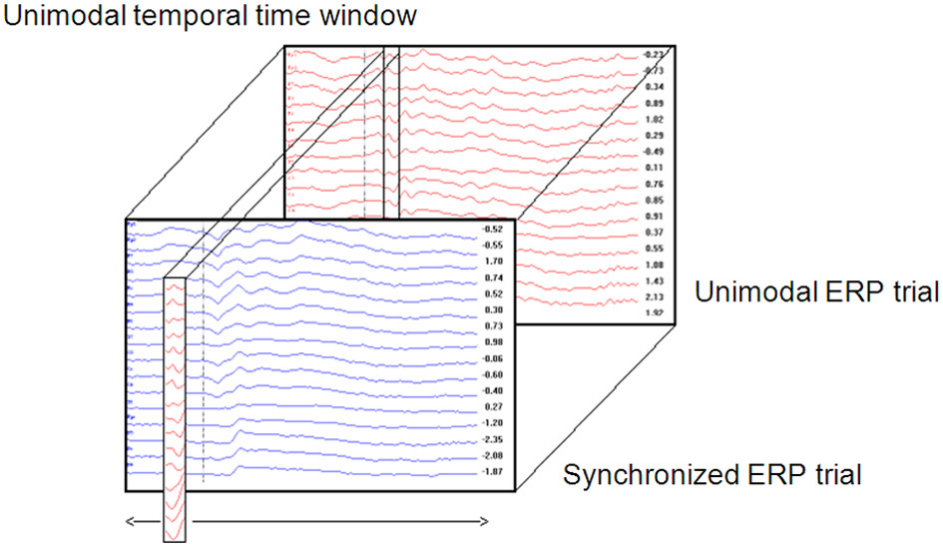
**Cross-correlation analysis between a unimodal time window throughout the crossmodaltrial**.

**Figure 2:**
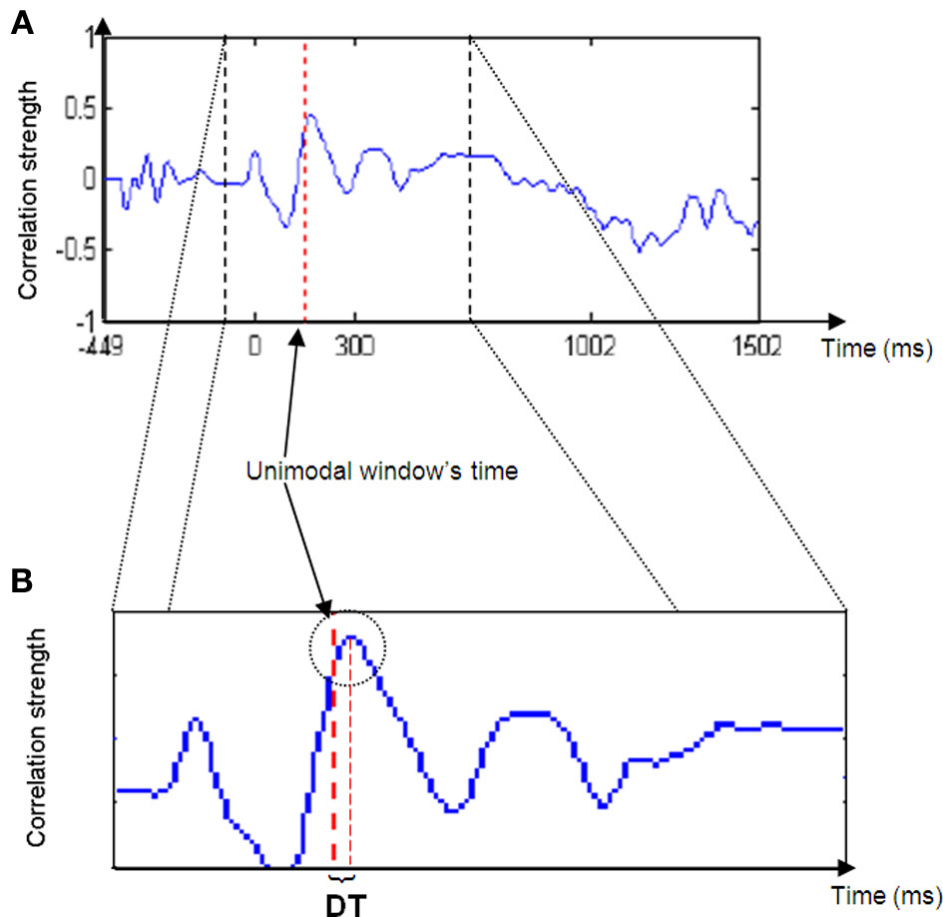
**Cross-correlation strength graph.** Note the peak in graph strength in the area of the unimodal time window (right red dashed line in **B**) and the delta time (DT) between the line and the peak maximum.

1. A unimodal time window was constructed by taking the values (amplitude) of all electrodes from the unimodal dataset across a certain time window (Figure 1). Thus, a time window is defined as a two-dimensional dataset with electrodes as rows and time samples as columns. The time window's location is defined as the center of the specific time window. For example, a time window taken at the time area of 150 ms is defined as the amplitude data of all electrodes between 100 and 200 ms (see Temporal implementation of Phases A and B for an explanation of the choice of time window location).
2. A cross-correlation loop between the above time window and the crossmodal ERP was applied as follows:
  - A crossmodal ERP time window of the same duration of the unimodal ERP time window constructed in Phase 1 was computed. The first crossmodal ERP time window used in the algorithm was from 0 to 100 ms. Note that the algorithm takes into account a time window of all electrodes altogether.
  - The correlation strength between the two time windows was calculated and resulted in a number within the range of −1 and 1. A value of zero indicates no correlation. A correlation value that approaches −1 indicates a strong negative correlation and a value that approached 1 indicates a strong positive correlation.
  - A new crossmodal ERP time window was then constructed which was located one successive time frame (3.9 ms) after the crossmodal ERP time window constructed in Phase 2a.
  - This procedure was repeated until the end of the crossmodal ERP trial duration (see Figure 1).
3. The result of the algorithm is a series of correlation coefficients computed for each unimodal ERP time window location every 10 ms, from the beginning of the ERP trial to the end.
  If assumption 1 is correct, then the cross-correlation graph should appear as in Figure 2.
  Thus, for most of the iterations (Phases 2b to 2d) the correlation between the unimodal ERP window and the crossmodal ERP should be relatively low, but at a particular time, the correlation strength should increase and reach a relatively high peak. The peak's location is the point in time at which the ERP data of the unimodal window have their maximum similarity to the crossmodal ERP. This notion can be understood as two pictures that look almost the same.
4. The DT between the peak time and the center of the unimodal window was measured. DT is the parameter used as the basis for the next phase of analysis.

### Delta time (DT) parameter

The dependent variable in the proposed algorithm consists of the DT between the unimodal window's location and the correlation peak's time location. If the unimodal time window represents a temporal fraction of information processing and there is a relatively strong correlation between the window and a window similar in size from the crossmodal ERP, then the algorithm suggests that the same temporal fraction of information processing occurred in both ERPs. In addition, if there is a DT between the peak location and the unimodal window, then the fraction of information processing occurred earlier or later, depending upon the DT polarity. Thus, a negative DT indicates early occurrence of the information processing fraction, or in other words, the second modality influenced and accelerated the process of the first modality. In contrast, a positive DT indicates late occurrence of the information processing fraction, which means that the second modality decelerated the SOP of the first modality.

The algorithm found the DT for each participant between the peak's location and the center of the unimodal time window. A *t*-test assessed whether there was a significant difference on DT values between the two groups of participants.

### Temporal implementation of phases A and B

The phases described above (A and B) focus on a particular time window (for example, between 100 and 200 ms). However, SOP rate can change throughout time. In addition, it is reasonable to assume that the second modality's degree of influence is varied at different time locations. Therefore, the last phase of the algorithm runs across the entire unimodal time course. In other words, the DT for each time window's location for each participant was calculated between the location of the unimodal window and the location of the correlation peak with the crossmodal ERP. For each of the time windows, an average DT was computed for each group. It has been suggested (Goswami, 2011) that dyslexic individuals suffer from difficulties at processing information presented at rates corresponding to the occurrence of syllabic information in speech, and critical for speech comprehension (4–7 Hz, Giraud and Poeppel, 2012). Information at these frequencies arrive to the brain every 142–250 ms. Therefore, it is reasonable to assume that in the current study, where the task was to react to a single event stimulus (visual, auditory, or crossmodal type), the DT of each of the groups would differ statistically within this time area. To verify this assumption, a series of *t*-test analyses was run on the DT parameter (that was computed based on each of the time windows taken from the time area of 140–250 ms) to assess whether there was a significant difference between the two groups.

### Implementation of phase C on both modalities

**Figure 3:**
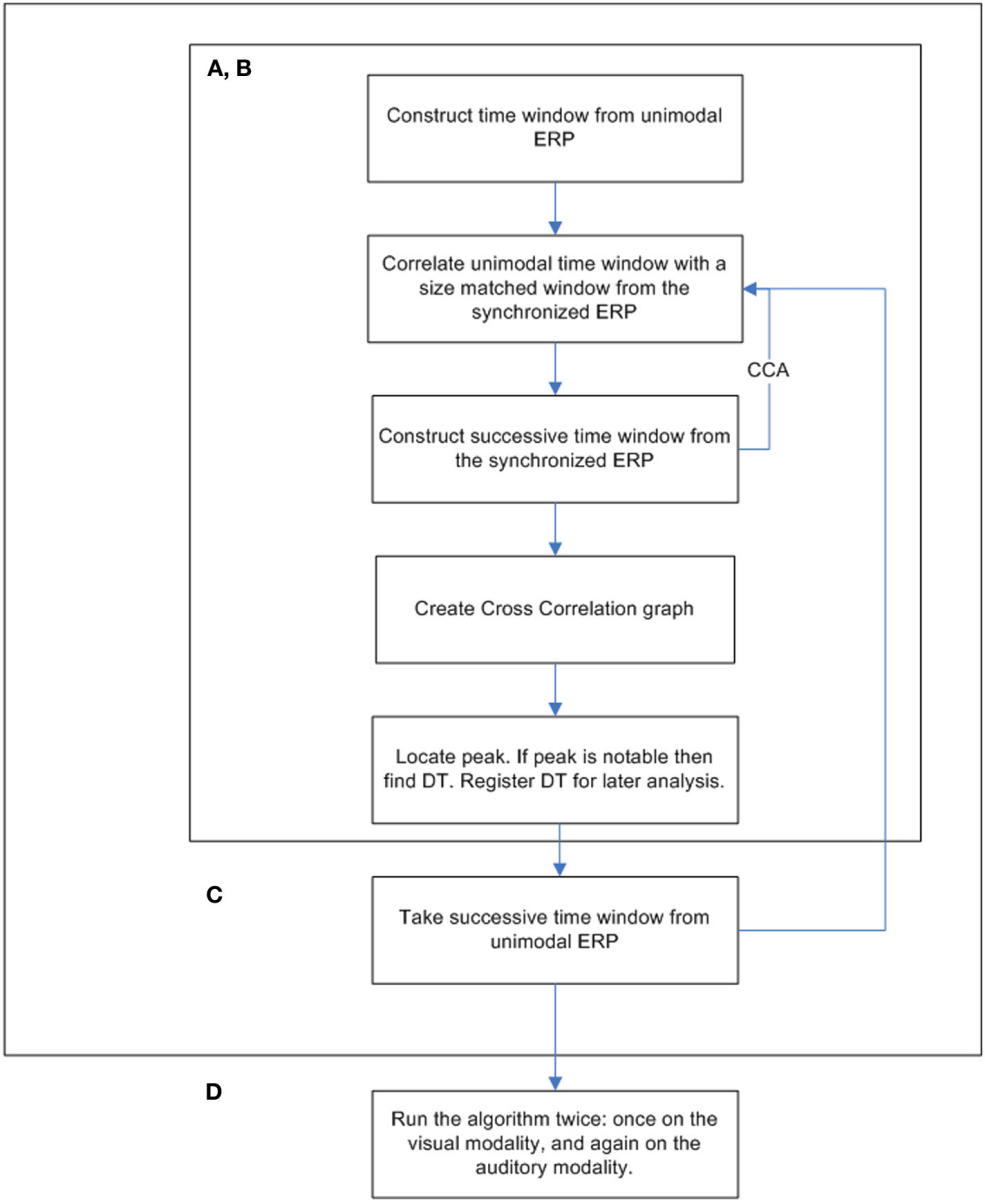
**The cross-correlation algorithm flow chart**.

The procedure was run twice, once for each modality. The window locations were set between 50 and 650 ms. Each successive time window was moved in increments of 10 ms (i.e., the second time span was centered at 60 ms, the third at 70 ms, etc.; see Figure 3 for a full flowchart description of the cross-correlation algorithm).

## Results

### Experimental behavioral measures:

In order to investigate task accuracy and RT when processing visual, auditory and crossmodal integration, an analysis of variance (ANOVA) in a 2 × 3 design (Group (dyslexic Xnon-impaired readers) × conditions (visual-only × auditory-only × crossmodal) was employed for RT and Accuracy separately. No significant between-group differences were found in accuracy [*F*_(1, 34)_ = 2.48, *p* > 0.05]. A significant Condition effect was obtained [*F*_(2, 68)_ = 50.2, *p* < 0.001] which stemmed from a lower performance under the auditory only condition for both groups (Table 2). No significant group by Condition interaction was found [*F*_(2, 68)_ = 0.22, *p* > 0.05].

**Table 2:** **Mean (and Standard Deviation) for the dyslexic and non-impaired readers of the behavioral reading and experimental measures**.

	**Dyslexic readers**	**Non-impaired readers**	***t***	***p***
Visual correct responses (%)	73 (20.8)	83 (16)	1.43	n.s
Auditory correct responses (%)	65 (19.5)	73 (16.3)	1.02	n.s
Visual + Auditory correct responses (%)	71.8 (18.7)	83.3 (14.2)	1.68	n.s
Visual reaction time (ms)	669 (115)	565 (122)	2.23	<0.05
Auditory reaction time (ms)	692 (110)	586 (129)	2.29	<0.05
Visual + auditory reaction time (ms)	639 (136)	587 (66)	1.12	n.s

The analysis of the RT data revealed a significant group effect [*F*_(1, 34)_ = 4.45, *p* < 0.05], but no significant condition effect [*F*_(2, 68)_ = 1.2, *p* > 0.05] and no significant group by condition interaction [*F*_(2, 68)_ = 1.44, *p* > 0.05].

### ERP cross-correlation analysis results

In order to investigate the influence of the presence of one modality on the SOP of the other modality, several cross-correlation analyses were obtained on averaged on-going ERP. The first analysis was obtained in order to investigate the influence of the auditory modality on the SOP of the visual one and the second analysis investigated the influence the visual modality on SOP of the auditory one. The cross-correlation analysis was run between sequential time windows taken from each unimodal condition and the crossmodal condition. The cross-correlation analysis outcome measure, the visual and auditory DT, was then used to compare between-modalities' influence and between group differences.

**Figure 4:**
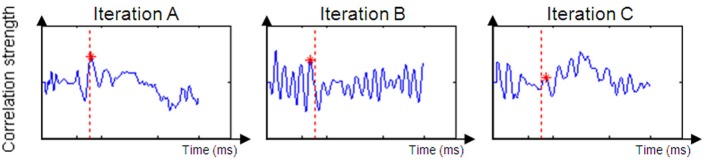
**Examples of a successful correlation result (Iteration A) and unsuccessful correlation result (Iterations B and C).** Iteration **(A)** produced a positive peak near the original time of the unimodal window. Iteration **(B)**'s dataset is too noisy to suppose that the value of DT is reliable. Iteration **(C)**'s peak is to low and expresses a weak correlation between the two datasets.

Based on the size of the correlation between the unimodal data and the crossmodal ERP data within an allotted time frame, it can be inferred that a given time window from the unimodal condition exists in the crossmodal ERP data (see Figure 4).

**Figure 5:**
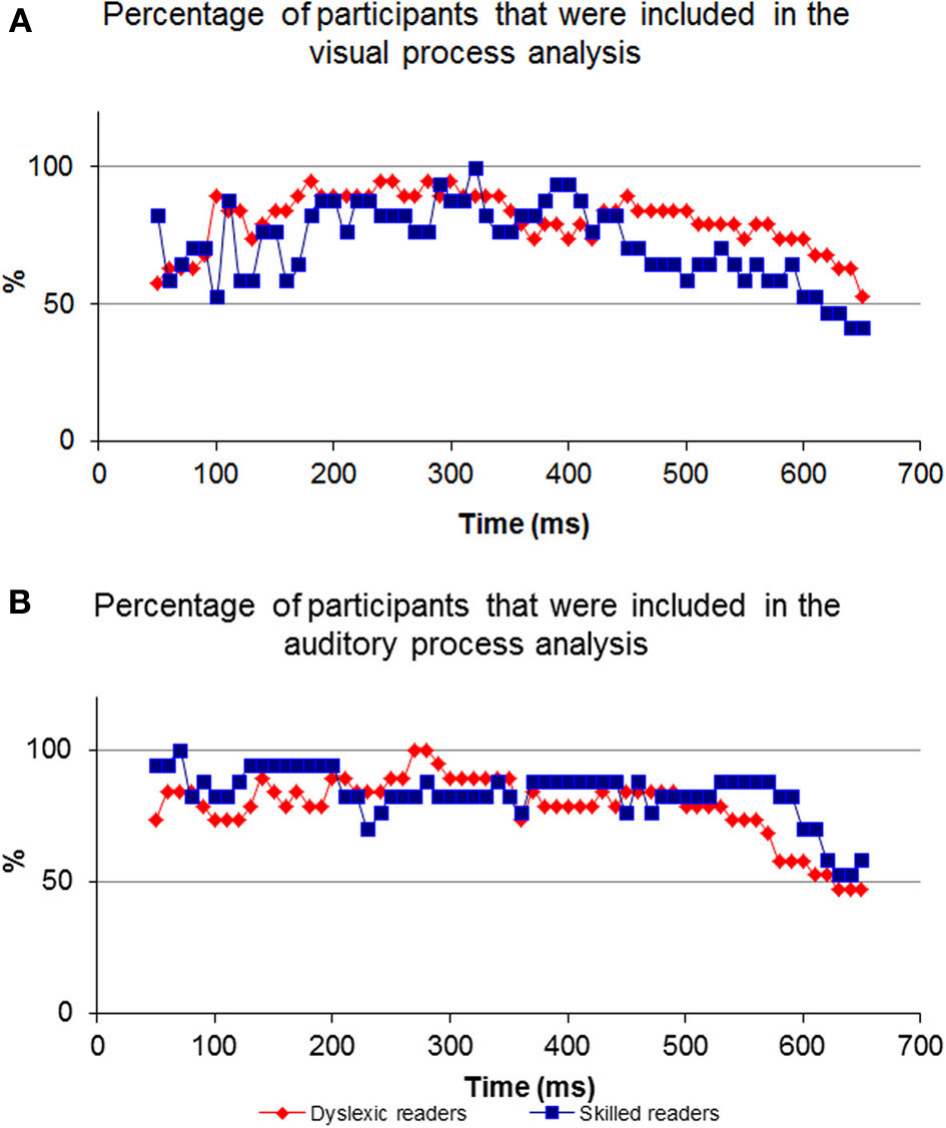
**The percentage of participants included in every comparison.** Note the inverted ‘U’ shape of the visual process graph where at the beginning and end of the process the percentage of participants is relatively low compared to the middle. In contrast to the visual process's graph, the auditory graph is stable at the beginning.

For most participants, a notable peak could be identified (see Figure 4A), though results should be interpreted with caution as the graph's shape did not always indicate successful correlation strength (see Figures 4B,C). For example, iteration B's dataset is too noisy to determine the peak's location because of an artificial waveform and iteration C's is very local and low. Therefore, inclusion conditions were developed and applied: only cross-correlation graphs which had a peak which was located no more than 50 ms before or after the time of the unimodal window were included in the computation of the DT average. The time window total width of 100 ms (50 ms before and after the time window location) was based on previous evidence that asserted that the variance of ERP component time locations is normally distributed in a time area of less than 50 ms before and after the component mean time location (for example, see Simon et al., 2007; Maurer et al., 2008; Spironelli and Angrilli, 2009). In addition, the graphs could not contain more than 3 additional local peaks within the given time window. These criteria kept distorted datasets out of the further analysis (see Phase B). Figure 5 shows the percentage of participants included in the process in every time window location. Figures 5A,B reveal the percentage of participants included in each time window analysis throughout the visual and auditory information process analysis, respectively. Overall, a relatively low number of participants from both groups were included in the visual analysis in the time areas of 50–150 and 450 ms to the end (Figure 5A). It is interesting to note that toward the end of the trial, the percentage of dyslexic readers included in the analysis decreased more slowly than the percentage of included non-impaired readers. In the auditory analysis (Figure 5B), the percentage rate of included participants in both groups remained constant from the beginning of the trial until about 500 ms.

**Figure 6:**
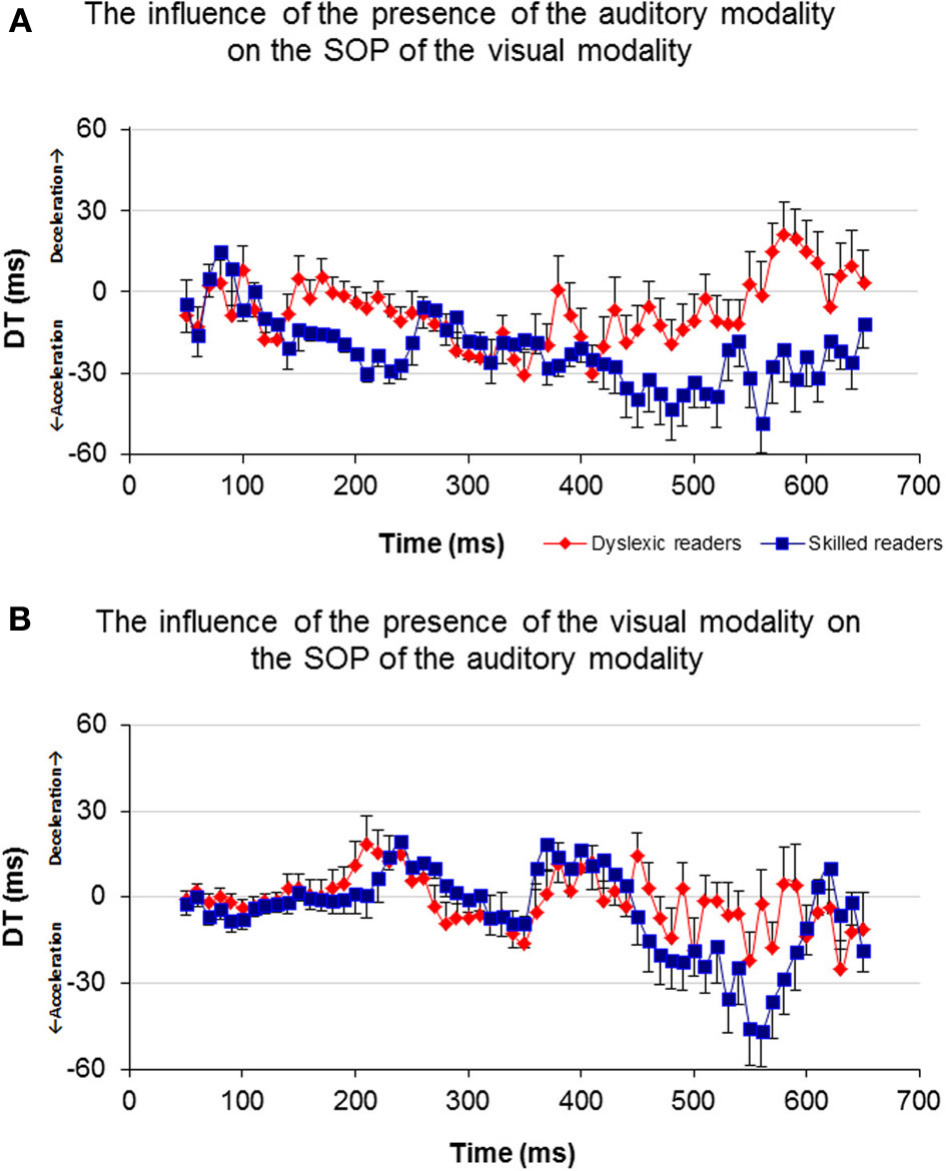
**Average DT of the two groups for each time window.** Axis X represents time and axis Y represents DT. **(A)** The DT results of the visual-only and the crossmodal ERPs. **(B)** The DT results of the auditory-only and the crossmodal ERPs.

In order to investigate the influence of one modality's activation on the SOP of the other modality, a DT for each of the participants and for each of the time windows was computed (Figure 6). A *t*-test analysis was applied for time windows located within the time area of 140–250 ms (Table 3), to determine if there was a between-group difference in each of the time windows (it is important to note here that a *t-test* was used to assess between-group differences rather than repeated measures analysis of variance (rmANOVA) due to the number of degrees of freedom and the relatively insufficient number of participants in this study). When the DT was based on the influence of the auditory modality on the SOP of the visual modality, significant between-group differences were found in the time area of 170 ms through 240 ms (Figure 6A, Table 3). In contrast, no significant between-group differences were found when the DT was based on the influence of the visual modality on the SOP of the auditory modality (Figure 6B). Nevertheless, note the positive peak (de-acceleration) in the value of the non-impaired readers' DT in the time area of 240 ms, and of the dyslexic readers' similarly shaped peak occurring at 210 ms.

**Table 3:** **The comparison between the groups' DT computed from the Visual modality time windows taken from the time area of 140–250 ms**.

**Window location (ms)**	***t***_(35)_	***p***
140	1.157	0.258
150	1.595	0.122
160	1.426	0.167
170	2.506	0.019
180	2.553	0.016
190	2.872	0.007
200	3.033	0.005
210	3.645	0.001
220	3.022	0.005
230	2.7	0.011
240	2.199	0.036
250	1.022	0.315

## Discussion

The purpose of this experiment was to investigate the effect of one modality on the SOP of the second modality. Stimuli of visual and auditory modalities were employed in a unimodal and crossmodal presentation. Overall, our results support the notion that there is in fact an interaction between modalities during information processing, consistent with previous research. Furthermore, the significant differences that were found between dyslexic and non-impaired readers highlight the difficulty which dyslexic readers face in synchronizing processing between the visual and auditory modalities.

Hypothesis 1 suggested that the ERP data of the crossmodal condition would contain unimodal visual and auditory elements. It is suggested that the peak in the correlation graph results supports this assumption (Figure 2A). As described previously, a cross-correlation analysis computing successive correlation coefficients between unimodal ERP within a specific time window and successive crossmodal ERP time windows sliding along the entire crossmodal trial was implemented. More specifically, the current algorithm took a fraction (i.e., 100 ms) of the temporal information processing from a unimodal ERP trial and searched for a matching fraction of information processing throughout the crossmodal ERP trial. It is apparent from Figure 2A that throughout the sequence, the correlation strength was mainly low and randomized. However, at a particular point in time, an increase in the correlation strength appeared, usually a short distance in time from the time location of the unimodal window. It may be argued that this constitutes evidence for the presence of the same fraction of information processing that occurs in the crossmodal ERP. A short while after the correlation strength reaches its peak, it starts to decline as the unimodal window passes its identical fraction of information processing and continues toward the time area in which low and randomized similarity appear.

Hypothesis 2 implied that the presence of a second modality will have an effect on the SOP of a first modality. Figure 2B reveals that the correlation graph's peak location is not in the exact location of the unimodal time window. Furthermore, data analysis across the entire time domain and across participants revealed that the correlation peak location may appear shortly before or after the unimodal window's time location. It is suggested that the time difference between the two locations (i.e., DT) is a reflection of the degree of influence of the second modality on the first modality's SOP when presented simultaneously. If the closest matching fraction of information processing taken from a unimodal ERP was found at a later time location (i.e., DT is positive), then this comparison indicates that there was a delay in the processing of the modality in the crossmodal ERP, meaning that SOP decelerated. Conversely, if the DT was negative, then the location of the same fraction of information processing existed at an earlier point of time in the crossmodal ERP compared to the unimodal ERP. Consequently, this constitutes evidence of SOP acceleration in the modality.

The neurobiology of the visual system is considered to be constructed from two major pathways (the dorsal and ventral), where visual information enters the retina and travels to the visual cortex. Information reaches the visual cortex about 100–150 ms post visual stimulus presentation. Auditory processing begins at the ear and initial auditory information reaches the auditory cortex through several pathways about 70–100 ms post stimulus presentation (see Breznitz, 2006 for review). Both modality pathways prepare initial sensory information for higher cognitive processing. As the pathways process information rapidly and in areas deep within the brain, it is expected that their operations will not have a strong impact on EEG data. Therefore, comparing the visual-only ERP and the crossmodal ERP during the first 150 ms of data collection will result in a relatively lower “success” rate when locating the visual information processing fraction in the crossmodal ERP (Figure 5A). However, as time progresses and processing moves from deeper brain areas to higher cortical structures, the size of the common components in the two ERP sets increases. Therefore, the algorithm has more success in finding common visual components 150 ms post stimulus presentation. As a result, a higher percentage of participants are included in the analysis from 150–450 ms. When finding the common components between the auditory-only ERP and crossmodal ERP, a higher “success” rate was found at an earlier time frame (50 ms post stimulus presentation) as initial auditory processing is faster than initial visual processing (Figure 5B).

Shifting our focus to the end of processing within the visual modality, a differential between the dyslexic reading group and non-impaired readers is apparent beginning at around 450 ms post stimulus presentation (Figure 5A). The differential begins at this point in time due to the decrease in the percentage of non-impaired readers included in the analysis. The percentage of dyslexic readers included in the analysis remains high for a longer period of time. The decrease in the percentage rate of included participants for analysis implies that among the non-impaired readers, the shared elements of the two ERPs end at that point. Thus, the duration of visual information processing is approximately 450 ms, and, as such, the ERP data appearing after 450 ms no longer deals with visual information, which results in a lower “success” rate of finding a sufficient correlation between the two datasets. However, visual information processing lasts longer in the dyslexic group and as such, the percentage of participants starts to decrease approximately 50 ms later. This is an important difference between the two groups as it provides evidence for slow speed of visual processing among the dyslexic readers as compared to non-impaired readers.

As can be seen in Figure 6A, non-impaired readers' visual processing “benefits” from the presence of the auditory synchronized stimulus as the value of DT is negative almost throughout the entire timeline (i.e., their SOP of visual information is accelerated). A similar but lower effect was observed among the dyslexic readers. Furthermore, a significant difference was found between the values of DT appearing around 170 and 240 ms where the DT of the non-impaired readers was more negative than that of the dyslexic readers. This time area is considered to be related to perception and has been suggested by previous research to be related to the dyslexia phenomenon (Maurer et al., 2006, 2008). On the other hand, processing within the auditory modality was not affected by the presence of a visual stimulus appearing from about 50–200 ms in both groups (Figure 6B). The non-impaired readers began to decelerate their auditory SOP around 210 ms. Similar deceleration was observed among the dyslexic readers but 40 ms earlier. Based on the reasoning that visual processing is slower than auditory processing, we provide additional evidence that in the normal information processing sequence, whenever there is a need to synchronize the two modalities, the brain accelerates processing within the visual modality and decelerates processing within the auditory modality. Unlike the non-impaired readers, the dyslexic readers do not accelerate their visual SOP to the same degree (Figure 6A). Moreover, they decelerated their auditory processing too early (Figure 6B). It is possible that this leads to asynchrony within the two modalities and to an overall slowness of information processing.

Failure at the level of lower sensory processing, which was the focus of the present study, may accumulate in the higher order levels of processing such as processing letters, syllables, words, sentences, and general reading comprehension. As reading activates sequential parallel visual and auditory processes, synchronization between the two modalities is necessary for successful reading accuracy and rate. The results obtained in the current study concerning common elements between single modality and crossmodal processing lend support to the synchronization hypothesis (Breznitz and Misra, 2003).

Prior studies in the field of temporal processing have been focused on the ability of the brain to process input at different frequencies (Buzsaki and Draghun, 2004; Luo and Poeppel, 2007; Power et al., 2012). Specifically, the temporal sampling framework of dyslexia (Goswami, 2011) suggests that the dyslexic reader may suffer from atypical processing of information occurring at frequencies between 4 and 7 Hz, i.e., every 142–250 ms (theta band, and possibly lower frequencies). The current results indicate an apparent failure of dyslexic readers in processing information occurring within the specific time area of 150–250 ms following a single event stimulus (Figure 6). The non-impaired readers demonstrated a non-symmetric effect of one modality on the other, in which the occurrence of bimodal information processing accelerated the SOP of the visual modality and decelerated the SOP of the auditory modality at the time area of 170–250 ms. This concurrent change in the two modalities' SOP was not obtained among the dyslexic readers as their visual modality's SOP did not accelerate in the presence of auditory information processing (Figure 6). It could be that, as proposed by the temporal sampling framework of dyslexia (Goswami, 2011), atypical neural oscillations at the theta frequency band for auditory processing among the dyslexic readers impact negatively the SOP of their visual modality processing, by preventing it from accelerating adequately. Thus, it may be suggested that these results provide additional support for the difficulty of the dyslexic individual to process a 4–7 Hz inflow of information and moreover, support the temporal sampling framework of dyslexia (Goswami, 2011; Power et al., 2012).

### Conflict of interest statement

The author declares that the research was conducted in the absence of any commercial or financial relationships that could be construed as a potential conflict of interest.

## References

[B1] Adams M. J. (1990). Beginning to Read: Learning and Thinking About Print. Cambridge, MA: MIT Press

[B2] Amitay S. Ben-Yehuda G. Banai K. Ahissar M. (2002a). Disabled readers suffer from visual and auditory impairments but not from a specific magnocellular deficit. Brain 125, 2272 10.1093/brain/awf231 12244084

[B3] Amitay S. Ben-Yehudah G. Nelken I. (2002b). Auditory processing deficits in reading disabled adults. J. Assoc. Res. Otolaryngol. 3, 302 10.1007/s101620010093 12382105 PMC3202414

[B4] Badian N. A. (1997). Dyslexia and the double deficit hypothesis. Ann. Dyslexia 47, 69 10.1007/s11881-997-0021-y

[B5] Barker T. A. Torgeson J. K. Wagner R. K. (1992). The role of orthographic processing skills on five different reading tasks. Read. Res. Q. 335 10.2307/747673

[B6] Ben-Artzi E. Marks L. E. (1995). Visual-auditory interaction in speeded classification: role of stimulus difference. Percept. Psychophys. 57, 1151 10.3758/BF03208371 8539090

[B7] Ben-Yehudah G. Ahissar M. (2004). Sequential spatial frequency discrimination is consistently impaired among adult dyslexics. Vision Res. 44, 1047 10.1016/j.visres.2003.12.001 15031099

[B8] Berninger V. (1990). Multiple orthographic codes: key to alternative instructional methodologies for developing the orthographic-phonological connections underlying word identification. School Psych. Rev. 19, 518

[B9] Berninger V. (2001). Understanding the ‘Lexia'in dyslexia: a multidisciplinary team approach to learning disabilities. Ann. Dyslexia 51, 21 10.1007/s11881-001-0004-3

[B10] Botuck S. Turkewitz G. (1990). Intersensory functioning: auditory visual pattern equivalence in younger and older children. Dev. Psychol. 26, 115 10.1037/0012-1649.26.1.115

[B11] Breznitz Z. (2001). The determinants of reading fluency: a comparison of dyslexic and average readers, in Dyslexia, Fluency and the Brain, ed Wolf M. (Timonium, MD: York Press), 245–276

[B12] Breznitz Z. (2002). Asynchrony of visual-orthographic and auditory-phonological word recognition processes: an underlying factor in dyslexia. Read. Writ. 15, 15 10.1023/A:1013864203452

[B13] Breznitz Z. (2003). Speed of phonological and orthographic processing as factors in dyslexia: electrophysiological evidence. Genetic Soc. Gen. Psychol. Monogr. 129, 183–206 14606733

[B15] Breznitz Z. (2006). Fluency in Reading: Synchronization of Brain Processes. Mahwah: Lawrence Erlbaum Associates

[B16] Breznitz Z. (2008). Special issue on the use of electrophysiological measures in reading research. J. Neurolinguistics 21, 277 10.1016/j.jneuroling.2007.02.001

[B17] Breznitz Z. Misra M. (2003). Speed of processing of the visual–orthographic and auditory–phonological systems in adult dyslexics: The contribution of “asynchrony” to word recognition deficits. Brain Lang. 85, 486 10.1016/S0093-934X(03)00071-3 12744959

[B83] Breznitz Z. Shaul S. Horowitz-Kraus T. Sela I. Nevat M. Karni A. (2013). Enhanced reading by training with imposed time-constraint in typical and dyslexic adults. Nat. Commun. 4:1486 10.1038/ncomms2488 23403586

[B86] Brickenkamp R. (1981). Test d2: Aufmerk-sankeits-delastungf-test. Gottingen: Varlag fur Psychology

[B80] British Psychological Society. (1999). Dyslexia literacy and psychological assessment, in Report by a Working Party of the Division of Educational and Child Psychology (Leicester: British Psychological Society). 10.1002/dys.181 11305231

[B18] Bruck M. (1992). Persistence of dyslexics' phonological awareness deficits. Dev. Psychol. 28, 874–886 10.1037/0012-1649.28.5.874

[B19] Buzsaki G. Draghun A. (2004). Neuronal oscillations in cortical networks. Science 304, 1926–1929 10.1126/science.1099745 15218136

[B20] Chen Q. Zhou X. (2013). Vision dominates at the preresponse level and audition dominates at the response level in cross-modal interaction: behavioral and neural evidence. J. Neurosci. 33, 7109–7121 10.1523/JNEUROSCI.1985-12.2013 23616521 PMC6619562

[B21] Cunningham A. E. Stanovich K. E. (1990). Assessing print exposure and orthographic processing skill in children: a quick measure of reading experience. J. Educ. Psychol. 82, 733 10.1037/0022-0663.82.4.733

[B22] Egeth H. E. Sager L. C. (1977). On the locus of visual dominance (vision over audition). Percept. Psychophys. 22, 77 10.3758/BF03206083

[B24] Fujisaki W. Nahida S. (2005). Temporal frequency characteristics of synchrony–asynchrony discrimination of audio-visual signals. Exp. Brain Res. 166, 455–464 10.1007/s00221-005-2385-8 16032402

[B25] Ghajar J. Ivry R. B. (2008). The predictive brain state: asynchrony in disorders of attention? Neurorehabil. Neural Repair 22, 217–227 10.1177/1545968308315600 18460693 PMC4338277

[B26] Ghitza O. Greenberg S. (2009). On the possible role of brain rhythms in speech perception: intelligibility of time-compressed speech with periodic and aperiodic insertions of silence. Phonetica 66, 113–126 10.1159/000208934 19390234

[B27] Giraud A. L. Poeppel D. (2012). Cortical oscillations and speech processing: emerging computational principles and operations. Nat. Neurosci. 15, 511–517 10.1038/nn.3063 22426255 PMC4461038

[B28] Goswami U. Gerson D. Astruc L. (2010). Amplitude envelope perception, phonology and prosodic sensitivity in children with developmental dyslexia. Read. Writ. 23, 995–1019 d 10.1007/s11145-009-9186-6

[B29] Goswami U. (2011). A temporal sampling framework for developmental dyslexia. Trends Cogn. Sci. 15, 3–10 10.1016/j.tics.2010.10.001 21093350

[B30] Groth K. Lachmann T. Riecker A. Muthmann I. Steinbrink C. (2011). Developmental dyslexics show deficits in the processing of temporal auditory information in German vowel length discrimination. Read. Writ. 24, 285–303 10.1007/s11145-009-9213-7

[B31] Heil M. Rolke B. Engelkamp J. Roesler F. Oezcan M. Hennighausen E. (1999). Event-related brain potentials during recognition of ordinary and bizarre action phrases following verbal and subject-performed encoding conditions. Eur. J. Cogn. Psychol. 11, 261 10.1080/713752313

[B32] Hulme C. Goetz K. Gooch D. Adams J. Snowling M. J. (2007). Paired-associate learning phoneme awareness, and learning to read. J. Exp. Child Psychol. 96, 150–166 10.1016/j.jecp.2006.09.002 17145064

[B33] Jasper H. H. (1958). The 10-20 electrode system of the international federation. Electroencephalogr. Clin. Neurophysiol. Evoked Potentials I, 371 10590970

[B34] Jones M. W. Branigan H. P. Parra M. A. Logie R. H. (2013). Cross-modal binding in developmental dyslexia. J. Exp. Psychol. Learn. Mem. Cogn. 39, 1807–1822 10.1037/a0033334 23773185

[B35] Koppen C. Levitan C. A. Spencer C. (2009). A signal detection study of the Colavita visual dominance effect. Exp. Brain Res. 196, 353–360 10.1007/s00221-009-1853-y 19488743

[B36] Lachmann T. Berti S. Kujala T. Schroger E. (2005). Diagnostic subgroups of developmental dyslexia have different deficits in neural processing of tones and phonemes. Int. J. Psychophysiol. 56, 105–120 10.1016/j.ijpsycho.2004.11.005 15804446

[B37] Leonard C. Eckert M. Lombardino L. J. Oakland T. Kranzler J. Mohr C. M. (2001). Anatomical risk factors for phonological dyslexia. Cerebral Cortex 11, 148–157 10.1093/cercor/11.2.148 11208669

[B38] Liberman I. Y. Shankweiler D. (1991). Phonology and beginning to read: a tutorial, in Learning to Read: Basic Research and its Implications, eds Reiben L. Perfetti C. A. (Hillsdale, NJ: Lawrence Erlbaum Associates), 3–18

[B39] Livingstone M. S. (1991). Physiological and anatomical evidence for a magnocellular defect in developmental dyslexia. Proc. Natl. Acad. Sci. U.S.A. 88, 7943 10.1073/pnas.88.18.7943 1896444 PMC52421

[B40] Llinas R. (1993). Is dyslexia a dyschronia? Ann. N.Y. Acad. Sci. 682, 48 10.1111/j.1749-6632.1993.tb22958.x 8323159

[B41] Luo H. Poeppel D. (2007). Phase patterns of neuronal responses reliably discriminate speech in human auditory cortex. Neuron 54, 1001–1010 10.1016/j.neuron.2007.06.004 17582338 PMC2703451

[B42] Lyon G. R. Shaywitz S. E. (2003). Defining dyslexia, comorbidity,teachers' knowledge of lLanguage and reading: a definition of dyslexia. Ann. Dyslexia 53, 1–14 10.1007/s11881-003-0001-9

[B43] Marchant J. L. Driver J. (2013). Visual and audiovisual effects of isochronous timing on visual perception and brain activity. Cereb. Cortex 23, 1290–1298 10.1093/cercor/bhs095 22508766 PMC3643713

[B87] MATAL Battery. (2007). Diagnostic Battery for the Assessment of Learning Functions. Jerusalem: The Council for Higher Education and the Center for Psychometric Tests

[B44] Maurer U. Brem S. Brem F. Kranz K. Bucher R. Benz P. (2006). Coarse neural tuning for print peaks when children learn to read. Neuroimage, 33, 749–758 10.1016/j.neuroimage.2006.06.025 16920367

[B45] Maurer U. Rossion B. McCandliss B. D. (2008). Category specificity in early perception: face and word N170 responses differ in both lateralization and habituation properties. Front. Hum. Neurosci. 2:18 10.3389/neuro.09.018.2008 19129939 PMC2614860

[B47] Melara R. D. O'Brien T. P. (1987). Interaction between synesthetically corresponding dimensions. J. Exp. Psychol. 116, 323 2522534

[B48] Meyler A. Breznitz Z. (2003). Processing of phonological, orthographic and cross-modal word representations among adult dyslexic and normal readers. Read. Writ. 16, 785 10.3200/GNTP.166.2.215-240 15906933

[B49] Mishra J. Martinez A. Sejnowski T. J. Hillyard S. A. (2007). Early cross-modal interactions in auditory and visual cortex underlie a sound-induced visual illusion. J. Neurosci. 27, 4120–4131 10.1523/JNEUROSCI.4912-06.2007 17428990 PMC2905511

[B50] Morris J. S. Ohman A. Dolan R. J. (1998). Conscious and unconscious emotional learning in the human amygdala. Nature 393, 467–470 10.1038/30976 9624001

[B52] Neil W. Roach N. W. Heron J. Whitaker D. McGraw P. V. (2011). Asynchrony adaptation reveals neural population code for audio-visual timing Proc. R. Soc. B 278, 1314–1322 10.1098/rspb.2010.1737 20961905 PMC3061136

[B53] Nelson-Wong E. Howarth S. Winter D. Callaghan J. (2009). Application of autocorrelation and cross-correlation analyses in human movement and rehabilitation research. J. Orthop. Sports Phys. Ther. 39, 287–295 10.2519/jospt.2009.2969 19346626

[B46] Orgil Medical Equipment. (1997). Analysis Software User's Manual for the Brain Performance Measurement System. Ein Ayala

[B54] Posner M. I. Nissen M. J. Klein R. M. (1976). Visual dominance: an information-processing account of its origins and significance. Psychol. Rev. 83, 157 10.1037/0033-295X.83.2.157 769017

[B55] Power A. J. Mead N. Bames L. Goswami U. (2012). Neural entrainment to rhythmically presented auditory, visual, and audio-visual speech in children. Front. Psychol. 3:216 10.3389/fpsyg.2012.00216 22833726 PMC3400256

[B56] Rosenzweig M. R. Bennet E. L. (1996). Psychobiology of plasticity: effects of training and experience on brain and behavior. Behav. Brain Res. 78, 57 10.1016/0166-4328(95)00216-2 8793038

[B57] Saito D. N. Yoshimura K. Kochiyama T. Okada T. Honda M. Sadato N. (2005). Cross-modal binding and activated attentional networks during audio-visual speech integration: a functional MRI study. Cereb. Cortex 15, 1750–1760 10.1093/cercor/bhi052 15716468

[B58] Schmolesky M. T. Wang Y. Hanes D. P. Thompson K. G. Leutgeb S. Schall J. D. (1998). Signal timing across the macaque visual system. J. Neurophysiol. 79, 3272–3278 9636126 10.1152/jn.1998.79.6.3272

[B59] Sela I. Breznitz S. Breznitz Z. (2008). The correlation-based model: An alternative system for analyzing ERP data in cognitive research. J. Neurlinguistics 21, 305–332 10.1016/j.jneuroling.2007.07.003

[B60] Share D. L. (1994). Deficient phonological processing in disabled readers implicates processing deficits beyond the phonological module, in Current Directions in Dyslexia Research, eds van den Bos K. P. Siegel L. S. Bakker D. J. Share D. L. (Lisse: Swets and Zeitlinger), 149–167

[B61] Shaywitz S. Shaywitz B. (2008). Paying attention to reading: the neurobiology of reading and dyslexia. Dev. Psychopathol. 20, 1329–1349 10.1017/S0954579408000631 18838044

[B62] Simon G. Rebai M. Petit L. Bernard C. (2007). N170 ERPs could represent a logographic processing strategy in visual word recognition. Behav. Brain Func. 3, 21 10.1186/1744-9081-3-21 17451598 PMC1884163

[B63] Snowling M. J. (1995). Phonological processing and developmental dyslexia. J. Res. Read. 18, 132–138 10.1111/j.1467-9817.1995.tb00079.x

[B64] Spironelli C. Angrilli A. (2009). Developmental aspects of automatic word processing: language lateralization of early ERP components in children, young adults and middle-aged subjects. Biol. Psychol. 35–45 10.1016/j.biopsycho.2008.01.012 18343558

[B65] Stanovich K. E. (1988). Explaining the differences between the dyslexic and the garden-variety poor reader: the phonological-core variable-difference model. J. Learn. Disabil. 21, 590–612 10.1177/002221948802101003 2465364

[B66] Stanovich K. E. West R. F. (1989). Exposure to print and orthographic processing. Read. Res. Q. 24, 402 10.2307/747605

[B67] Stein J. (1993). Dyslexia-impaired temporal information processing? Ann. N.Y. Acad. Sci. 682, 83–86 10.1111/j.1749-6632.1993.tb22961.x 8323162

[B68] Stein J. (2001). The magnocellular theory of developmental dyslexia. Dyslexia 7, 12 10.1002/dys.186 11305228

[B81] Stein J. Talcott J. (1999). Impaired neuronal timing in developmental dyslexia—the magnocellular hypothesis. Dyslexia 5, 59–77 10.1002/(SICI)1099-0909(199906)5:2<59::AID-DYS134>3.0.CO;2-F

[B69] Stein J. Talcott J. Witton C. (2001). The sensorimotor basis of developmental dyslexia, in Dyslexia: Theory and Good Practice, ed Fawcett A. J. (London: Whurr), 65–88

[B82] Stein J. Walsh V. (1997). To see but not to read; the magnocellular theory of dyslexia. Trends Neurosci. 20, 147–152 10.1016/S0166-2236(96)01005-3 9106353

[B70] Tallal P. (1980). Language and reading: some perceptual prerequisites. Bull. Orton Soc. 30, 170 10.1007/BF02653716

[B71] Tallal P. Merzenich M. M. Miller S. Jenkins W. (1998). Language learning impairments: Integrating basic science, technology, and remediation. Exp. Brain Res. 123, 210 10.1007/s002210050563 9835411

[B72] Tallal P. Miller S. Fitch R. S. (1993). Neurobiological basis of speech: a case for the preeminence of temporal processing. Ann. N.Y. Acad. Sci. 682, 27 10.1111/j.1749-6632.1993.tb22957.x 7686725

[B73] Temple E. Poldrack R. A. Salidis J. Deutsch G. K. Tallal P. Merzenich M. M. (2001). Disrupted neural responses to phonological and orthographic processing in dyslexic children: An fMRI study. Neuroreport 12, 299–307 10.1097/00001756-200102120-00024 11209939

[B74] Vellutino F. R. Fletcher J. M. Snowling M. J. Scanlon D. M. (2004). Specific reading disability (dyslexia): What have we learned in the past four decades? J. Child Psychol. Psychiatry 45, 2 10.1046/j.0021-9630.2003.00305.x 14959801

[B75] Wechsler D. (1997). WAIS-III Administration and Scoring Manual. San Antonio, TX, The Psychological Corporation

[B77] Wolf M. Bowers P. (2000). The question of naming-speed deficits in developmental reading disability: an introduction to the Double-Deficit Hypothesis. J. Learn. Disabil. 33, 322–324 10.1177/002221940003300404 15493094

[B78] Woody C. D. (1967). Characterization of an adaptive filter for the analysis of variable latency neuroelectric signals. Med. Biol. Eng. 5, 539 10.1007/BF02474247

[B79] Zecker S. G. (1991). The orthographic code: developmental trends in reading-disabled and normally-achieving children. Ann. Dyslexia 41, 178 10.1007/BF02648085 24233764

